# Philadelphia Dispensary

**Published:** 1839-03-30

**Authors:** 


### PHILADELPHIA DISPENSARY.

*Report of Cases treated from the* 1*st to the* 28*th of February*, *inclusive.*

| | | |
|---|---|---|
| Whole number . . . | | 182 |
| Discharged cured . . . | 143 | |
| Dead . . . . . . | 3 | |
| Sent to the Almshouse . . | 1 | |
| Remaining under treatment . | 35—182 | |

Among the deaths, were from

| | |
|---|---|
| Hydrocephalus . . . . . . | 2 |
| Gastritis . . . . . . . . | 1 |
| Total . . . . . . . | 3 |

